# Efficacy of External Beam Radiation-Based Treatment plus Locoregional Therapy for Hepatocellular Carcinoma Associated with Portal Vein Tumor Thrombosis

**DOI:** 10.1155/2016/6017406

**Published:** 2016-11-24

**Authors:** Ming-Yang Chen, Yu-Chao Wang, Tsung-Han Wu, Chen-Fang Lee, Ting-Jung Wu, Hong-Shiue Chou, Ngan-Ming Tsang, Kun-Ming Chan, Wei-Chen Lee

**Affiliations:** ^1^Department of General Surgery, Chang Gung Memorial Hospital at Linkou, Chang Gung University College of Medicine, Taoyuan, Taiwan; ^2^Department of Radiation Oncology, Chang Gung Memorial Hospital at Linkou, Chang Gung University College of Medicine, Taoyuan, Taiwan; ^3^Division of Liver and Transplantation Surgery, Department of General Surgery, Chang Gung Memorial Hospital at Linkou, Chang Gung University College of Medicine, Taoyuan, Taiwan

## Abstract

*Background*. Portal vein tumor thrombosis (PVTT) is a common event in advanced hepatocellular carcinoma (HCC). The optimal treatment for these patients remains controversial.* Methods*. A retrospective review of 149 patients who had unresectable HCC associated with PVTT between January 2005 and December 2012 was performed. Outcomes related to external beam radiation-based treatment were measured, and clinicopathological features and parameters affecting prognosis were analyzed as well.* Results*. The radiotherapeutic response of PVTT was an important element that affected the overall treatment response of HCC. Serum *α*-fetoprotein < 400 ng/mL, the presence of a radiotherapeutic response on PVTT, and receiving additional locoregional therapy were significant prognostic factors affecting the survival of patients. Patients who had received additional locoregional therapy obtained a better outcome, and six of them were eventually able to undergo surgical management with curative intent.* Conclusion*. The outcome of HCC associated with PVTT remains pessimistic. In addition to the current recommended treatment using sorafenib, a combination of external beam radiotherapy targeting PVTT and locoregional therapy for intrahepatic HCC might be a promising strategy for patients who had unresectable HCC with PVTT. This approach could perhaps offer patients a favorable outcome as well as a possible cure with following surgical management.

## 1. Introduction

Hepatocellular carcinoma (HCC) is a globally common and lethal malignancy. In addition to its high prevalence, early diagnosis of HCC remains poor despite aggressive surveillance programs. As a result, most patients are diagnosed with advanced HCC, which is characterized by larger tumor size, multiple nodules, or vascular invasion and distant metastasis [1, 2]. With regard to vascular invasion, portal vein tumor thrombosis (PVTT) accounts for the most common presentation associated with locally advanced HCC. It is estimated that 10 to 40% patients with HCC have PVTT at the time of diagnosis; the prognosis of such patients is extremely poor because of limited treatment options [3–5].

Although an aggressive surgical approach for advanced HCC associated with PVTT could provide a favorable prognosis in selected patients, the long-term outcome of surgical treatment for these patients is still unsatisfactory [6–8]. Moreover, patients who are eligible for surgical resection are small number, whereas a great number of patients with HCC and PVTT have no optimal treatment options. The overall survival is approximately 2 to 4 months for patients without any treatment for HCC concurrent with PVTT [9, 10]. As such, various modalities including transarterial chemoembolization (TACE), radioembolization, hepatic artery infusion chemotherapy (HAIC), external beam radiotherapy, or a combination of the above modalities have been attempted for unresectable HCC associated with PVTT.

Among these modalities, radiotherapy focused on PVTT seems to be reasonable as it could deliver a local therapeutic effect on PVTT in HCC patients. In this investigation, we gathered data on patients who had advanced HCC with PVTT to evaluate the therapeutic outcome and prognosis of external beam radiotherapy focused on PVTT. Meanwhile, the feasibility and outcome of radiotherapy combined with other modalities for these patients were assessed as well.

## 2. Materials and Methods

### 2.1. Patients

Patients who had received external beam radiotherapy for primary HCC at the Department of Radiotherapy, Chang Gung Memorial Hospital at Linkou in Taiwan, during the period from January 2005 to December 2012 were retrospectively reviewed. Under the approval of the Institutional Review Board, a total of 645 registered patients who had unresectable HCC were thoroughly reviewed using medical records. Among these, patients who had unresectable HCC associated with PVTT and liver function with a Child-Pugh score ≤ 6 were included in this study. Patients with the presence of extrahepatic metastasis, a Child-Pugh score beyond 7 (Child B or C cirrhosis), or tumor thrombosis extending to the main hepatic vein and/or inferior vena cava (IVC) at the time of radiotherapy were excluded from the present analysis. Finally, 149 patients (116 men and 33 women) with ages ranging from 20.6 to 93.8 years (median age 59.7 years) were included in the study.

### 2.2. Diagnosis and Treatment of HCC

The diagnosis of HCC was based on the diagnostic guidelines by the European Association for the Study of the Liver (EASL) and the American Association for the Study of Liver Diseases (AASLD) [11, 12]. Generally, the measurement of serum *α*-fetoprotein (AFP) and imaging examination of either dynamic liver computed tomography (CT) or magnetic resonance imaging (MRI) are mandatory. In addition, liver tumor biopsy was considered only in cases of an equivocal imaging pattern that was inadequate to establish diagnosis or if it was clinically indicated. The degree and extent of PVTT were determined based on the imaging examination and classified according to the classification system proposed by Liver Cancer Study Group of Japan [13]. Briefly, the level of PVTT was classified into four categories: Vp1, PVTT confined to distal portal branches; Vp2, PVTT extended to second-order portal branches; Vp3, PVTT involved in first-order portal branches; and Vp4, PVTT detected in the main portal trunk.

The treatment of HCC was based on the consensus obtained from the members of the liver cancer committee, comprising liver surgeons, oncologists, hepatologists, diagnostic radiologists, and interventional radiologists. The treatment modality consisted of multidisciplinary therapy, including surgical treatment, locoregional therapy by TACE and/or radiofrequency ablation (RFA) or percutaneous ethanol injection (PEI), radiotherapy, chemotherapy, or a combination of these treatments. The selection of treatment modality mainly depended on the balance of three factors including tumor characteristics, liver function reserve, and the patient's physical condition. Liver resection was always the preferred treatment method whenever the tumor was considered to be resectable based on the radiologic examination and the liver function reserve. However, liver resection was ineligible for patients with evidence of extrahepatic metastasis, main portal trunk and/or inferior vena cava invasion, and multiple HCCs with both hepatic lobes involvement. Apart from that, the extent of liver resection was determined according to the Makuuchi algorithm [14], and patients with poor liver function reserve reflected by high retention rate of indocyanine green (ICG) test were also unsuitable for liver resection [1, 15].

### 2.3. Radiotherapy and Evaluation

In general, external beam radiotherapy is used for symptom control of patients with advanced HCC as a palliative treatment or for local disease control of patients with liver-confined HCC that are not eligible for other therapies such as liver resection and locoregional therapies. Meanwhile, external beam radiotherapy is also used as an extra treatment combined with other modalities to achieve a better therapeutic response.

External beam radiotherapy using three-dimensional conformal radiotherapy (3DCRT) or intensity-modulated radiotherapy (IMRT) was planned after the identification of PVTT and was performed mainly targeting on the defined PVTT area. The liver tumor was not included in the irradiation area unless the primary hepatic tumor was close to the defined PVTT area. With these modern forms of radiotherapy, 3DCRT and IMRT provide highly conformal dose distributions in the target volumes and minimize the dose received by adjacent structures.

The therapeutic response of HCC was evaluated every three months or whenever medical need was indicated. Assessments were performed by dynamic liver CT according to the modified Response Evaluation Criteria in Solid Tumor (mRECIST) criteria [16]. Generally, a complete response (CR) was represented by the disappearance of arterial enhancement in all target lesions, and a partial response (PR) was defined by at least a 30% decrease in the sum of the diameters of viable target lesions. An increase of at least 20% in the sum of the diameters of viable target lesions was considered progressive disease (PD), and any cases that did not qualify for either PR or PD were considered stable disease (SD). The best therapeutic responses were evaluated in terms of PVTT and HCC status. The assessment of the PVTT response was mainly focused on the portal vein. However, the overall HCC response was a result of the combined assessment of target lesions, nontarget lesions, and new lesions.

### 2.4. Outcomes and Statistical Analysis

Outcomes were measured in terms of overall survival (OS), which was estimated from the date of HCC and PVTT diagnosis until the date of death or last follow-up. Survival curves were compared by the log-rank test and constructed using the Kaplan-Meier method. The clinical variables between the different groups were examined using the chi-square or Fisher's exact test as appropriate. Prognostic variables were analyzed using the Cox regression proportional hazards model to identify factors influencing outcomes, and all prognostic factors determined to be significant from the univariate analysis were then selected for the multivariate analysis. All data were analyzed using the Statistical Package for the Social Sciences (SPSS) version 20.0 (SPSS, Inc., Chicago, IL) for Windows. A *p* value <0.05 was considered statistically significant.

## 3. Results

### 3.1. Clinical Characteristics of Patients

The major etiology of HCC was hepatitis B virus infection, which accounted for 55.0% of patients; 77.8% of patients were male. Furthermore, 29 patients (19.5%) had previously undergone liver resection for primary HCC. Among these patients, three had received multiple liver resections for intrahepatic HCC. The presence of a tumor thrombus in the portal system occurred mainly at the portal branch (93 patients, 62.4%) that consisted of 61 Vp3, 31 Vp2, and 1 Vp1, whereas 56 patients (37.6%) had a tumor thrombus at the main portal trunk (Vp4). The median total irradiation dose was 33 Gy (range, 5–60 Gy). Overall, the response rate of PVTT was 23.5%, including CR or PR in 35 patients, while 81 patients (54.4%) showed SD or PD after the completion of radiotherapy. The PVTT response could not be obtained in 33 patients (22.1%) who died before the evaluation.

Based on the overall response of HCC, the clinical features of the patients with (CR + PR, *n* = 35) and without (SD + PD, *n* = 114) a response are summarized in Table 1. There were significant differences in hepatitis B and C virus infection rates, radiotherapeutic response of PVTT, and additional locoregional therapy between the two groups. Patients in the HCC response group had a higher percentage of radiotherapeutic response on PVTT (*p* < 0.001) and included a higher ratio of patients receiving additional locoregional therapy (*p* = 0.007).

### 3.2. Predictors of Therapeutic Outcome

Further detailed analyses regarding the prognostic factors for patient outcomes are summarized in Table 2. The univariate analysis showed that the size of the primary HCC, serum AFP level, tumor number, total radiation dose, radiotherapeutic response of PVTT, and additional locoregional therapy were significant factors. Subsequently, multivariate regression analysis of these factors indicated that serum AFP < 400 ng/mL, the presence of a radiotherapeutic response on PVTT, and receiving additional locoregional therapy were significant prognostic factors affecting the survival of patients.

### 3.3. Survival Analysis

**Figure 1:**
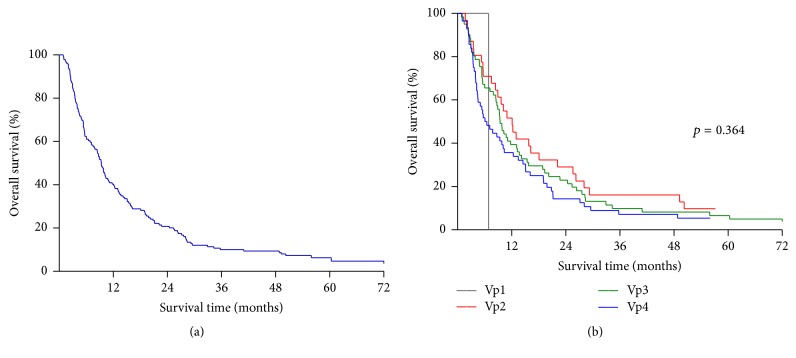
Kaplan-Meier survival curves of patients. (a) Overall cumulative survival curve of patients with hepatocellular carcinoma (HCC) and portal vein tumor thrombosis (PVTT). (b) Overall survival rates stratified by PVTT classification showed no significant difference. (*p* = 0.364) Vp1 (*n* = 1): PVTT in distal portal branches, Vp2 (*n* = 31): PVTT in second-order portal branches, Vp3 (*n* = 61): PVTT in first-order portal branches, Vp4 (*n* = 56): PVTT in the main portal trunk.

During the follow-up period, the median OS was 9.4 months, ranging from 0.9 to 123.9 months after the detection of PVTT. The overall 1-, 3-, and 5-year survival rates were 40.2%, 10.1%, and 6.3%, respectively (Figure 1(a)). The survival rates stratified by PVTT classification had no statistical difference, in which 1- and 3-year OS rates were both 0% for Vp1 (*n* = 1), 51.6% and 16.1% for Vp2 (*n* = 31), 39.3% and 9.8% for Vp3 (*n* = 61), and 35.7% and 7.1% for Vp4 (*n* = 56), respectively (Figure 1(b), *p* = 0.364).

**Figure 2:**
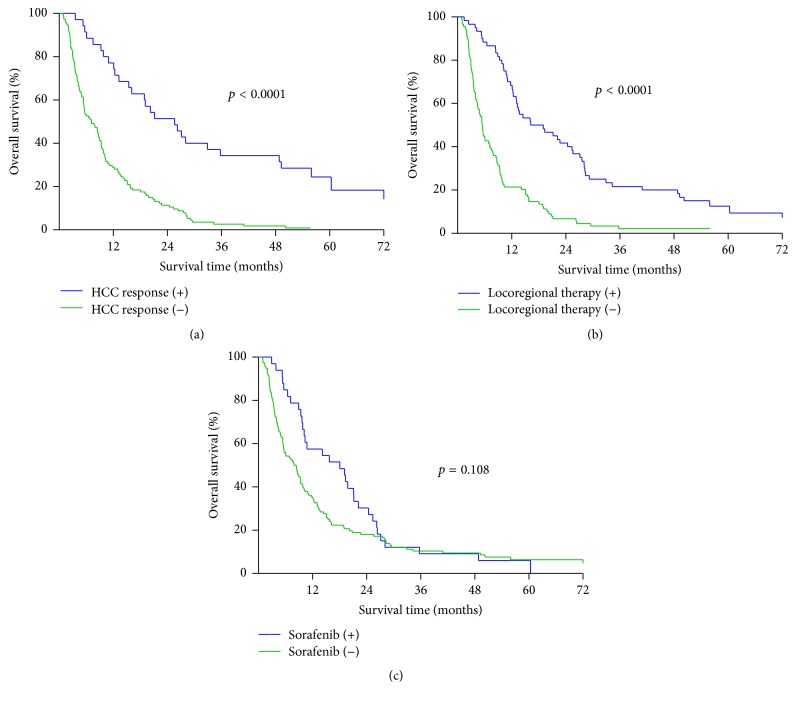
Comparison of survival rates regarding therapeutic responses and additional treatments. (a) Comparison of the overall therapeutic response of HCC; patients with a therapeutic response (CR, PR; *n* = 35) had a significant better survival curve than patients without a therapeutic response (SD, PD; *n* = 114) (*p* < 0.0001). CR: complete response, PR: partial response, SD: stable disease, and PD: progression disease. (b) Patients who had received additional locoregional therapy (*n* = 60) experienced significantly better survival as compared with patients who were unable to receive locoregional therapy (*n* = 89) (*p* < 0.0001). (c) No statistical difference in survival rates in terms of sorafenib treatment (*n* = 33) and no sorafenib treatment (*n* = 116) (*p* = 0.108).

The survival rates of patients who showed a treatment response of HCC were significantly better than those of patients without a response related to treatment (Figure 2(a), *p* < 0.0001). The 1-, 3-, and 5-year OS rates of the patients with a treatment response of HCC were 77.1%, 34.3%, and 18.4%, respectively, with a median survival of 25.6 months. The 1-, 3-, and 5-year OS rates of the patients without an HCC response related to treatment were 28.9%, 2.6%, and 0%, respectively, with a median survival of 7.2 months. Patients who had received additional locoregional therapy showed a better survival than patients who had no additional locoregional therapy (Figure 2(b), *p* < 0.0001). The 1-, 3-, and 5-year survival rates were 68.3%, 21.7%, and 12.5%, respectively, in patients with additional locoregional therapy (median survival 16.2 months). The 1- and 3-year survival rates were 21.3% and 2.2%, respectively, in patients without additional locoregional therapy (median survival 5.5 months). However, the outcome of these patients was not affected by whether they received sorafenib or not (Figure 2(c), *p* = 0.108). The 1-, 3-, and 5-year OS rates of patients given sorafenib versus those not given sorafenib were 57.6%, 9.1%, and 6.1% (median survival 18.1 months) versus 35.3%, 10.3%, and 6.4% (median survival 8.4 months), respectively.

Additionally, the six patients who had undergone surgical resection for HCC after the treatment of PVTT are described in Table 3. Of those, four patients received liver transplantation after downstaged HCC with complete remission of PVTT. Three of them were still alive and cancer-free at the end of this study, and one patient (patient 2) who had no HCC recurrence eventually died of HCV reactivation four years after transplantation. Two patients were eventually treated by liver resection: one (patient 4) showed PR of PVTT and SD of HCC after radiotherapy plus TACE and oral chemotherapy but had an HCC recurrence and died of cancer seven months after the operation; the other (patient 5) showed SD of PVTT and PR of HCC after radiotherapy plus TACE and died of postoperative hepatic failure 2.5 months after hepatectomy.

## 4. Discussion

Clinically, HCC has a strong tendency to invade the portal venous system, leading to formation of PVTT that could become a new source of cancer spread. The presence of PVTT is a common event in advanced HCC, and the optimal treatment for these patients remains largely controversial. Currently, multikinase inhibitors such as sorafenib are the only recommended treatment for HCC patients with PVTT, according to the Barcelona Clinic Liver Cancer (BCLC) staging system and therapeutic strategy [17, 18]. Nonetheless, the relatively low response rate and modest survival difference gained from previous reports regarding sorafenib are not optimistic. The recent improvement in radiotherapy techniques such as 3DCRT and IMRT has allowed for the delivery of a higher radiation dose to specific regions, and PVTT seems to be an entity that could be treated by this modality.

Historically, the liver is intolerable to irradiation, and thus external beam radiotherapy has limited use in HCC. However, recent advances in radiotherapy have strengthened its role as one of the major treatment modalities for HCC. A number of retrospective studies have shown a survival benefit of current radiotherapy in HCC patients with PVTT [19–21]. As shown by the present study, the radiologic response of PVTT was strongly connected with the overall therapeutic response of HCC and was reflected by the survival advantage as a significant prognostic factor. Nonetheless, despite the better outcomes seen in patients with a good therapeutic response, the general outcome of these patients remains dismal.

Previous studies have reported that the location of PVTT significantly influences the survival outcome of patients [9, 22, 23]. Theoretically, PVTT with main portal trunk involvement would decrease portal flow, leading to the elevation of portal pressure as well as the formation of varices, which would compromise liver functional reserve. Moreover, tumor thrombosis at the main portal trunk might possibly increase the risk of cancer spread. Taking these factors into account, PVTT at the trunk is associated with a poor prognosis in patients. However, the outcome of these patients was not affected by the location of PVTT in the present study. In contrast, the radiologic response of PVTT is much more important than the location, indicating that the treatment strategy remains crucial to patient outcomes.

Although radiotherapy is an optimistic modality for PVTT, the original intrahepatic HCC should also be properly managed in order to gain a better overall treatment effect. Among numerous modalities, locoregional therapy using either TACE or RFA/PEI has been a widely used tool for patients with inoperable HCC. However, TACE was historically contraindicated in HCC patients with PVTT for the sake of concerns regarding worsened liver dysfunction that might result from the embolic effect of TACE on an already compromised hepatic vascular supply. Recently, growing evidence has shown that intrahepatic artery superselective TACE can be performed safely in some patients with PVTT, leading to a better outcome [24–26]. Additionally, patients who have a good liver functional reserve and the presence of collateral circulation following PVTT can tolerate a modest delivery of TACE. Notably, our results show that locoregional therapy not only provides a survival benefit but also is an important prognostic factor, indicating that the combination of radiotherapy and additional locoregional therapy could significantly improve the overall outcome of HCC patients with PVTT.

Unlike other cancer diseases, HCC is relatively unsusceptible to traditional chemotherapeutic regimens. Currently, sorafenib is the only agent that has been shown to significantly improve survival in advanced HCC [17, 18]. Sorafenib has been found to modestly prolong survival in patients with macroscopic vascular invasion, presumably including PVTT as well. Nonetheless, the tumor response rates to sorafenib are disappointing, and the adverse reaction related to dosage and the cost has diminished its clinical benefit in certain patients. As shown by our data, only a small number of patients received sorafenib due to the high cost which was not covered by national health insurance before August 2012. Although the subgroup survival analysis regarding sorafenib showed no significant difference, the median survival of patients treated with sorafenib was 18.1 months, which is an improvement compared to 8.4 months of median survival in patients without sorafenib treatment. Therefore, future studies with a larger number of patients might be required to validate and demonstrate the survival benefit of sorafenib in patients with HCC and PVTT.

Additionally, the combination of external beam radiotherapy and/or other modalities such as HAIC and radioembolization have shown some potential benefit for patients with PVTT [27–30]. However, all of the above-mentioned modalities are considered to be palliative treatments, and surgical resection with curative intent remains the gold standard for HCC treatment because it offers the most favorable outcome. Although the long-term outcome of the surgical approach for advanced HCC associated with PVTT is still unsatisfactory, aggressive surgical resection could also be considered in selected patients due to the lack of better treatment options for these patients at the current time. Apart from that, the combination of radiotherapy and locoregional therapy as a bridge therapy prior to surgical resection with curative intent might be a promising strategy for patients with PVTT, as shown in this study. A few patients could perhaps enjoy long-term survival.

The major limitation of our study is that it was conducted in a single center with a small number of patients and was retrospective in nature. Although generalizations about the few reported cases could not be made easily, several marked differences might be helpful in managing patients with HCC and PVTT. Nonetheless, the argument that patients who tolerate additional locoregional therapy are naturally in a better condition than patients not receiving locoregional therapy could come across. Indeed, although HCC with PVTT is considered an advanced stage in the BCLC classification, patient in good clinical condition with better hepatic functional reserve and the presence of collateral circulation following PVTT might tolerate additional locoregional therapy and theoretically experience a better outcome. However, the accumulated data and this study have shown that an aggressive attitude using multidisciplinary treatment can effectively provide benefits to patients with HCC.

In summary, the overall outcome of patients with unresectable HCC complicated by PVTT remains pessimistic. The best therapeutic approach is still to be determined. Although sorafenib is now the only recommended treatment, additional radiotherapy combined with locoregional therapy such as TACE might be a promising alternative for HCC patients with PVTT. Moreover, a good response to radiotherapy for PVTT and the control of intrahepatic HCC by additional locoregional therapy could perhaps offer patients a favorable outcome as well as a cure with subsequent surgical management.

## Figures and Tables

**Table 1:** Clinical characteristics of patients with hepatocellular carcinoma and PVTT.

Characteristics	Therapeutic responses		*p* value
	CR, PR (*n* = 35)	SD, PD (*n* = 114)	
Age (years), median (range)	60 (36–78)	58 (21–94)	0.681
Male : female	27 : 8	89 : 25	0.908
Hepatitis B virus			0.041
Yes	14 (40.0%)	68 (59.6%)	
No	21 (60.0%)	46 (40.4%)	
Hepatitis C virus			0.003
Yes	20 (57.1%)	34 (29.8%)	
No	15 (42.9%)	80 (70.2%)	
Maximum tumor size (cm) Median (range)	5.0 (0.8–14.4)	5.6 (0.5–19.0)	0.951
AFP (ng/mL), median (range)	216.0 (2.0–33327.0)	294.0 (2.4–1183010)	0.062
Tumor number			0.808
Solitary	14 (40.0%)	43 (37.7%)	
Multiple	21 (60.0%)	71 (62.3%)	
Tumor distribution			0.856
Unilobar	23 (65.7%)	73 (64.0%)	
Bilobar	12 (34.3%)	41 (36.0%)	
Portal vein tumor thrombosis			0.053
Vp4	10 (28.6%)	46 (40.4%)	
Vp3	12 (34.3%)	49 (43.0%)	
Vp2	13 (37.1%)	18 (15.8%)	
Vp1	0	1 (0.8%)	
Duration of radiotherapy (days), median (range)	17 (4–44)	15 (1–52)	0.220
Total radiation dose (Gy), median (range)	39 (20–60)	30 (5–60)	0.097
Radiotherapeutic response of PVTT			<0.0001
CR, PR	21 (60.0%)	14 (12.3%)	
SD, PD	14 (40.0%)	67 (58.8%)	
N/A	0	33 (28.9%)	
Additional locoregional therapy			0.007
Yes	21 (60.0%)	39 (34.2%)	
No	14 (40.0%)	75 (65.8%)	
Chemotherapy			0.944
Sorafenib	8 (22.8%)	25 (21.9%)	
Other	10 (28.6%)	30 (26.3%)	
No	17 (48.6%)	59 (51.8%)	

CR: complete response; PR: partial response; SD: stable disease; PD: progression disease; AFP: alpha-fetoprotein; PVTT: portal vein tumor thrombosis; Vp1, PVTT in distal portal branches; Vp2, PVTT in second-order portal branches; Vp3, PVTT in first-order portal branches; Vp4, PVTT in the main portal trunk; N/A: not available.

**Table 2:** Univariate and multivariate analyses of clinicopathological factors affecting outcome of patients.

Factors	Univariate analysis			Multivariate analysis	
	*n*	Medium OS months (95% CI)	*p* value	HR (95% CI)	*p* value
Age (years)					
<55	50	7.1 (3.0–11.3)	0.560	—	
≥55	99	9.7 (7.9–11.5)			
Gender					
Male	116	8.9 (7.0–10.9)	0.823	—	
Female	33	9.4 (7.7–11.2)			
Hepatitis B virus					
Positive	82	8.9 (6.8–11.1)	0.299	—	
Negative	67	9.9 (5.7–14.0)			
Hepatitis C virus					
Positive	54	9.3 (4.9–13.7)	0.424	—	
Negative	95	9.4 (6.8–12.0)			
Maximum tumor size					
<10 cm	119	10.1 (7.7–12.5)	0.003	1	0.855
≥10 cm	30	4.0 (1.7–6.3)		1.04 (0.66–1.66)	
Serum AFP					
<400 ng/mL	80	11.7 (8.1–15.4)	0.008	1	0.016
≥400 ng/mL	69	6.1 (4.2–8.0)		1.52 (1.08–2.16)	
Tumor number					
Solitary	57	10.3 (7.4–13.1)	0.002	1	0.066
Multiple	92	8.5 (5.7–11.2)		1.41 (0.98–2.02)	
Distribution of primary HCC					
Unilobar	96	9.7 (7.4–11.9)	0.183	—	
Bilobar	53	9.3 (8.3–10.3)			
Portal vein thrombosis					
Main portal trunk	56	6.1 (3.1–9.1)	0.142	—	
Portal branch	93	9.9 (8.3–11.4)			
ECOG					
0,1	127	9.8 (8.4–11.2)	0.077	—	
≥2	22	5.3 (3.5–7.1)			
Total radiation dose					
<40 Gy	88	7.2 (4.3–10.1)	0.007	1.44 (0.99–2.10)	0.059
≥40 Gy	61	14.5 (8.9–20.1)		1	
Radiotherapeutic response of PVTT					
Yes	35	20.2 (12.5–28.0)	<0.0001	1	<0.0001
No	114	6.1 (3.6–8.6)		2.90 (1.85–4.56)	
Additional locoregional therapy					
Yes	60	16.3 (6.8–25.7)	<0.0001	1	<0.0001
No	89	5.5 (4.7–6.3)		2.65 (1.82–3.87)	
Kinases inhibitor					
Sorafenib	33	18.1 (8.4–27.7)	0.108	—	
No	116	7.9 (5.4–10.5)			
Additional chemotherapy					
Yes	53	10.1 (8.8–17.4)	0.940	—	
No	96	7.8 (4.8–10.8)			

OS: overall survival; CI: confidence interval; HR: hazard ratio; AFP: alpha-fetoprotein; HCC: hepatocellular carcinoma; ECOG: Eastern Cooperative Oncology Group; PVTT: portal vein tumor thrombosis.

**Table 3:** Clinical features of patient undergone surgical management with curative intent after radiotherapy and locoregional therapy.

Patient	Age/sex	Etiology	Maximum HCC	PVTT	Treatments	Radiotherapy of PVTT			Overall Responses	Surgical managements	Outcomes	
						Duration	Dose (Gy)	Response				
1	48 Y/M	HBV	2.0 cm	Left PV (Vp3)	Radiotherapy TACE/RFA	45 days	29	CR	CR	DDLT	123.9 ms	Alive
2	59 Y/M	HCV	4.5 cm	Right PV (Vp3)	Radiotherapy TACE	24 days	6	CR	CR	LDLT	77.5 ms	Death
3	49 Y/F	HBV	2.8 cm	Right PV (Vp2)	Radiotherapy TACE	45 days	20	CR	PR	LDLT	57.2 ms	Alive
4	77 Y/M	HBV	4.7 cm	Left PV (Vp3)	Radiotherapy TACE/UFT	35 days	13	SD	PR	Left hepatectomy	9.8 ms	Death
5	60 Y/M	No	6.0 cm	Right PV (Vp2)	Radiotherapy TACE	45 days	22	PR	SD	Extended right hepatectomy	12.26 ms	Death
6	51 Y/M	HBV	4.4 cm	Left PV (Vp2)	Radiotherapy TACE	45 days	21	CR	CR	DDLT	52.1 ms	Alive

Y: years old; M: male; F: female; HBV: hepatitis B virus; HCV: hepatitis C virus; HCC: hepatocellular carcinoma; PV: portal vein; PVT: portal vein tumor thrombosis; TACE: transcatheter arterial chemoembolization; RFA: radiofrequency ablation; UFT: tegafur & uracil; CR: complete response; PR: partial response; SD: stable disease; PD: progression disease; DDLT: decease donor liver transplantation; LDLT: living donor liver transplantation; ms: months.
